# Automated prediction of mastitis infection patterns in dairy herds using machine learning

**DOI:** 10.1038/s41598-020-61126-8

**Published:** 2020-03-09

**Authors:** Robert M. Hyde, Peter M. Down, Andrew J. Bradley, James E. Breen, Chris Hudson, Katharine A. Leach, Martin J. Green

**Affiliations:** 1 School of Veterinary Medicine and Science, University of Nottingham, Sutton Bonington Campus, Leicestershire, LE12 5RD United Kingdom; 2 Quality Milk Management Services, Cedar Barn, Easton Hill, Wells, BA5 1DU United Kingdom

**Keywords:** Computational biology and bioinformatics, Diseases, Medical research

## Abstract

Mastitis in dairy cattle is extremely costly both in economic and welfare terms and is one of the most significant drivers of antimicrobial usage in dairy cattle. A critical step in the prevention of mastitis is the diagnosis of the predominant route of transmission of pathogens into either contagious (CONT) or environmental (ENV), with environmental being further subdivided as transmission during either the nonlactating “dry” period (EDP) or lactating period (EL). Using data from 1000 farms, random forest algorithms were able to replicate the complex herd level diagnoses made by specialist veterinary clinicians with a high degree of accuracy. An accuracy of 98%, positive predictive value (PPV) of 86% and negative predictive value (NPV) of 99% was achieved for the diagnosis of CONT vs ENV (with CONT as a “positive” diagnosis), and an accuracy of 78%, PPV of 76% and NPV of 81% for the diagnosis of EDP vs EL (with EDP as a “positive” diagnosis). An accurate, automated mastitis diagnosis tool has great potential to aid non-specialist veterinary clinicians to make a rapid herd level diagnosis and promptly implement appropriate control measures for an extremely damaging disease in terms of animal health, productivity, welfare and antimicrobial use.

## Introduction

Mastitis is one of the most costly endemic diseases of dairy cattle^1^, being estimated to represent 38% of all direct production disease costs, with an estimated annual loss of £170 million in the UK^2^. In addition to substantial economic losses, mastitis is also a painful condition of detriment to animal welfare^3^, and has been shown to be one of the most significant drivers of antimicrobial usage in the UK dairy industry^4^.

Mastitis causing bacteria in cattle have historically been classified into two categories according to the main reservoirs and routes of infection; ‘contagious’ and ‘environmental’^5^. Contagious bacteria commonly exist within the mammary gland and are transmitted between cows during the milking process^6^. Environmental bacteria are not generally adapted to survive in the host but are opportunistic invaders from the cow’s environment. These are generally acquired between milking times and instigate an immune response rapidly dealt with by the immune system, resulting in a transient increase in white blood cells in milk. Since the control strategies for contagious mastitis differ markedly from those for environmental mastitis^7^, the ability to correctly diagnose the predominant transmission route of mastitis on farm is essential for successful implementation of control measures^8^.

In addition to differentiating mastitis of contagious and environmental origin, it is important to identify the time of the production cycle when the risk of new intramammary infections is highest. The nonlactating (“dry”) period has been shown to be at least as important as the lactating period in the epidemiology of intramammary infections^9^, and control strategies again differ between dry and lactation period origin mastitis^7^. The use of a categorical herd level mastitis diagnosis of either ‘environmental dry period’ (EDP), ‘environmental lactation period’ (EL) or ‘contagious’ (CONT) is one of the cornerstones of the AHDB Dairy Mastitis Control Plan (DMCP)^10^, an evidence based mastitis control programme applied in the UK^7^. The ability to correctly make this diagnosis is currently based on veterinary analysis of data recorded on farm, and requires considerable training, experience and specialist skills for data interpretation.

Machine learning (ML) classification algorithms have been used in a variety of applications, from the filtering of spam emails^11^ to the suggestion of movies a Netflix user might next enjoy^12,13^. Machine learning may also become an indispensable tool for medical clinicians, with algorithms approaching problems much like a medical clinician progressing through their clinical training might; learning rules from data and applying them to new patients^14^. The application of machine learning in the identification of disease has often focussed on image recognition, for example the accurate classification of skin cancer^15^, and retinal disease^16^. The use of machine learning techniques with diagnostic data, such as haematological results, has also been described, and were able to achieve an accuracy of 88% when compared with specialist haematologists; outperforming internal medicine specialists in achieving a correct diagnosis^17^. Machine learning has been used in the diagnosis of diabetes using features such as sex, age and blood pressure^18^, and the diagnosis of cardiac arrhythmia^19^, with random forest algorithms receiving particular attention in their ability to outperform many other machine learning algorithms in classification exercises^20^. Whilst machine learning has been able to provide accurate classification in the medical field, even successful predictions will have minimal impact on patient care without a collaborative approach between data scientists and clinicians to integrate the practice into real world settings^21^. Despite a large quantity of research in the use of machine learning to impact the clinical management of patients, examples of translation into an effect on clinical management are seldom found^22^.

ML has been used within the field of cattle medicine, for example in attempting to predict fertility outcomes^23^, high somatic cell counts^24^, and the onset of calving^25^. With the advent of increased “big data” within farm animal medicine, the potential to translate this into “smart data” is increasing^26^; making full use of data already being collected. Machine learning has been applied to epidemiological classification problems within cattle medicine, such as the prediction of bovine viral diarrhoea virus exposure at herd level^27^, and the distribution of exposure of herds to liver fluke^28^, and has recently been applied in the investigation of mastitis pathogen (*Streptococcus uberis*) transmission patterns in cattle^29^ as well as in the diagnosis of both subclinical^30,31^ and clinical^32^ mastitis at an individual animal level. Whilst machine learning has been described in both medical and veterinary fields and has been explored in the individual diagnosis of mastitis, it has not yet been applied to accurately replicate a specialist clinical diagnosis for a population level diagnosis, in this case the herd level diagnosis of bovine mastitis.

The aims of this study were to evaluate whether a complex, multifaceted specialist clinical decision could be replicated using machine learning algorithms. That is, in an instance in which a clinician had to synthesise and process multiple stands of information to reach a reasoned decision, it would potentially be possible to reach the same conclusion using machine learning methods with readily available farm management and disease data.

## Results

### Data source

Herd mastitis data were collected from 1,000 anonymised UK dairy herds via a milk recording company (QMMS Ltd) between 2009 and 2014. The data were randomly split into cross-validation (CV) and external validation (EV) sets and after filtering and pre-processing, a total of 278 farms and 290 farms were available for CV and EV (CONT vs ENV), and a total of 273 and 294 for CV and EV (EDP vs EL).

### Diagnosis of contagious or environmental infection patterns

#### Model parameter tuning

Model tuning and feature engineering were performed in a stepwise manner and accuracy, positive predictive value (PPV) and negative predictive value (NPV) were evaluated as model performance metrics. From a dense grid of values the optimal *mtry* (the number of variables randomly sampled as candidates at each split) was identified as 2 based on maximising accuracy.

Improvements in the model predicting contagious as opposed to environmental infection patterns were marginal through feature engineering and removal of poor quality data, with accuracy only increasing from 94.26% to 94.85%. Similarly, the use of recursive feature elimination did not have an important effect on model performance. Substantive model improvements were made by altering the classification threshold; the optimum threshold based on maximising accuracy was ≥0.35 for the diagnosis of CONT, which maximised PPV (100%) without detriment to NPV (95%).

#### Model performance

A high level of accuracy was achieved using Random forest algorithms to discriminate CONT vs ENV diagnoses. An accuracy of 95% was achieved from cross validation of the CV dataset, with a PPV and NPV of 100% and 95% respectively. Accuracy on external validation was similar at 98% with a PPV of 86% and NPV of 99% (with CONT as a “positive” diagnosis). A confusion matrix of external validation results are shown in Table 1, with a full description of performance metrics in Table 2.

**Table 1:** Confusion matrices for predictions of externally validated farms for the mastitis diagnoses made by specialist veterinary clinicians.

Observation						
Prediction		CONT	ENV		EDP	EL
	CONT	6	1	EDP	137	44
	ENV	4	279	EL	21	92

The diagnoses are described as contagious (CONT), environmental (ENV), environmental dry period (EDP) and environmental lactation period (EL).

**Table 2:** Performance metrics of externally validated farms for the mastitis diagnoses made by specialist veterinary clinicians.

	CONT vs ENV	EDP vs EL
Accuracy	98.28%	77.89%
Positive predictive value	85.71%	75.69%
Negative predictive value	98.59%	81.42%
Sensitivity	60.00%	86.71%
Specificity	99.64%	67.65%
F1 score	0.71	0.81
Kappa	0.69	0.55

The diagnoses are described as contagious (CONT), environmental (ENV), environmental dry period (EDP) and environmental lactation period (EL).

### Environmental dry period vs environmental lactation period

#### Model parameter tuning

**Figure 1:**
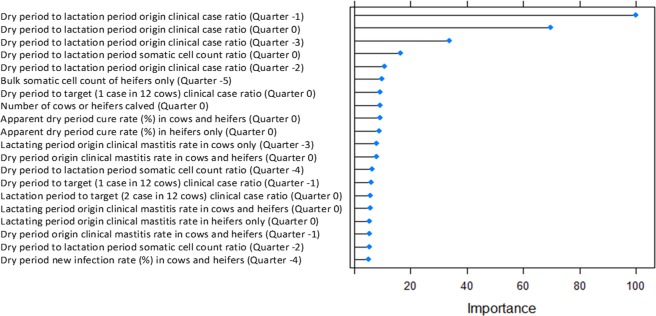
Variable importance plot showing the top 20 features for the classification of environmental dry period (EDP) vs environmental lactation period (EL) diagnosis. “Heifers” are animals in their first lactation, and “cows” are animals after their first lactation. “Quarter” denotes which 3-month period is being analysed; “Quarter 0” denotes the most recent 3-month period, and “Quarter -1” denotes the 3-month period prior to that.

From a dense grid of values the optimal *mtry* for the final model was identified as 61 based on maximising accuracy. Without any feature engineering, a 69.5% accuracy was achieved using the random forest algorithm to discriminate EDP from EL in the CV dataset. Additional aggregate features were engineered including the mean, median, sum, minimum and maximum values of all parameters across the data recording period. Ratio features were also created, including the ratio of mastitis cases <= 30 days in milk (the number of days post calving; DIM) to mastitis cases >30DIM. After feature engineering to create novel composite features an accuracy of 72.7% was achieved. Variable importance analysis illustrated that these new aggregate features were generally more important in classification of the mastitis diagnosis than the original features (Fig. 1).

**Figure 2:**
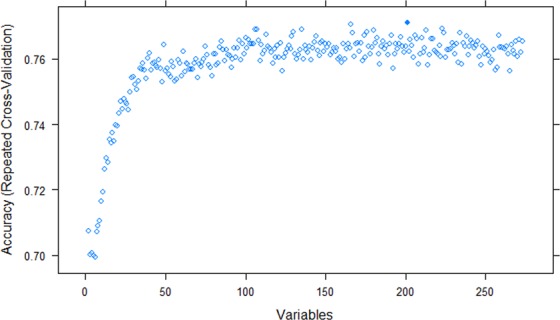
An illustration of the recursive feature elimination results incorporating 2 to 274 features (Random forest model to predict the herd mastitis diagnosis of environmental dry period or environmental lactation period). The number of features (variables) included within the model is depicted on the x-axis and the accuracy of the model from 10-fold cross validation on the truncated y-axis.

**Figure 3:**
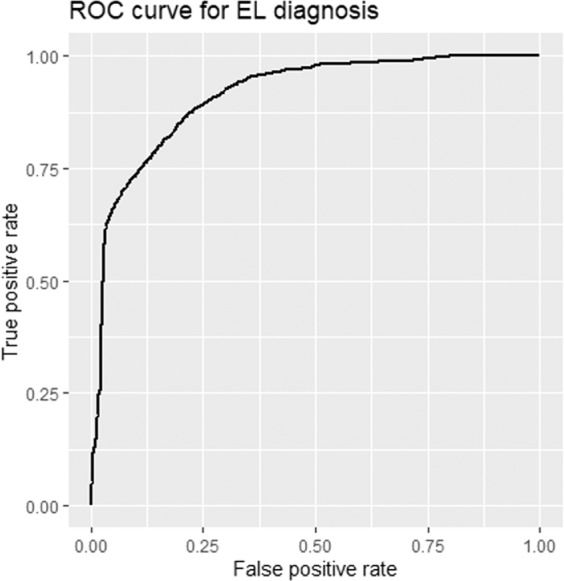
Receiver operator characteristic (ROC) curve of mastitis diagnosis of environmental lactation period origin (EL) as opposed to environmental dry period (EDP).

The removal of data rows with a large quantity (≥20%) of missing features, or where data quality was identified as likely to be poor at point of collection, resulted in improved model performance to an accuracy of 77.9% using the CV dataset. Recursive feature elimination (RFE) (Fig. 2) resulted in further improvements to model performance resulting in a final model accuracy on the CV dataset of 81%. Prediction thresholds for classification were examined, with CV predictions being performed using a threshold between 0 and 1 in 0.01 increments. Receiver operator characteristic (ROC) curves were also used to visually assess model performance (Fig. 3) and accuracy was used to determine the optimal threshold which was 0.50.

#### Final model performance

After feature engineering and selection, the model to predict a diagnosis of EDP or EL had an accuracy of 81% for the CV dataset, using 150 predictors, and a PPV and NPV of 81% and 82% respectively. External validation again resulted in similar performance with an accuracy of 78%, with a PPV of 76% and NPV of 81% respectively (with EDP as a “positive” diagnosis). A confusion matrix of external validation results are shown in Table 1, with a full description of performance metrics in Table 2.

### Calibration

**Figure 4:**
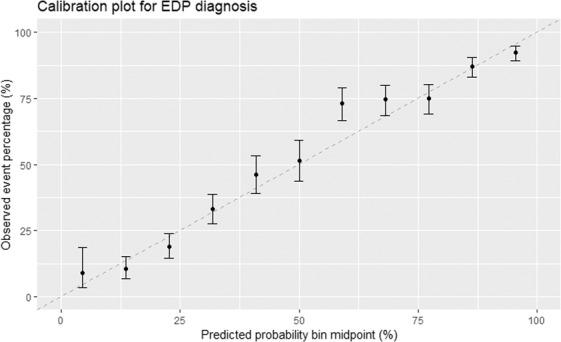
Calibration plot illustrating the predicted probability (and 95% confidence interval) of a mastitis diagnosis of environmental dry period origin (EDP), as opposed to environmental lactation period (EL), in comparison with the observed probability of the diagnosis.

The availability of accurate probability estimates for the automated diagnosis for clinicians was deemed extremely important. Calibration plots were used to evaluate the strength of the linear correlation between predicted and observed probabilities of each diagnosis (Fig. 4).

## Discussion

Using the random forest algorithm we were able to correctly replicate the herd level mastitis diagnosis with a high degree of accuracy when compared with a specialist veterinary clinician; using an external dataset, an accuracy of 98% was achieved for diagnosing CONT vs ENV and 78% for diagnosing EDP vs EL. This illustrates the potential for machine learning algorithms to reproduce the complex, specialist diagnosis of a veterinary clinician.

The diagnosis of herd level transmission patterns of mastitis on dairy farms is a critical step in implementing effective mastitis control strategies, however specialist training is required by the veterinarian to acquire these diagnostic skills. In addition, clinicians require a significant period of time dedicated to the analysis and interpretation of data in order to make the clinical diagnosis, which can be challenging in general practice. There is great potential for automated diagnostic support tools to reduce the amount of training and time required to make such a diagnosis; where a clinician might require 30–60 minutes to evaluate all the information and make a herd mastitis diagnosis, a machine learning algorithm requires only seconds. Such a convenient, rapid diagnostic test might consequently be performed more frequently than a veterinary clinician would manually, and therefore provide a more regular diagnostic report. This increased rate of diagnostic testing should provide earlier identification of changing patterns of mastitis and allow clinicians greater opportunity to focus on implementing mastitis control strategies than simply making the diagnosis itself.

Alongside the overall speed and predictive power of the algorithm, the predicted probability estimates are important in the clinical setting, and a 99% probability of a diagnosis would be interpreted differently to a 51% probability, despite both being attributed the same diagnoses. Calibration plots (Fig. 4) illustrate the observed event probability given a predicted probability; a ML diagnosis would be challenging to interpret in a clinical setting if a predicted probability of 80% only resulted in a correct diagnosis 20% of the time. The calibration plot “bins” predicted probabilities, plotting them against observed event percentages, with the desired outcome being a linear relationship between predicted and observed probabilities^33^. EDP vs EL calibration plots showed a strong association between predicted and observed probabilities and therefore by providing probability estimates of a diagnosis this would allow clinicians access to an accurate estimate of the uncertainty of a diagnosis rather than a binary, over-simplified outcome.

Further increases in the predictive accuracy of the machine learning algorithm to make the mastitis diagnosis may be achieved with increased granularity of farm data. The data for this study were exported from farms in aggregated 3-month periods, which may have resulted in some loss of information. The specialist veterinarian had access to monthly data from any time period, which may have made a difference in how data were interpreted. In this sense, clinicians had access to more data than the learning algorithms and it is certainly possible that predictive accuracy of ML could improve if these additional data were available. The specialist practitioner may also have had prior knowledge of farms that may have influenced the diagnosis made. This again would be unseen by the machine learning algorithm although does raise the question of whether prior knowledge could be incorporated in the prediction in a Bayesian setting.

Despite reporting a high predictive accuracy for external evaluation, for imbalanced datasets such as the CONT vs ENV diagnosis, there is the potential for these figures to be misleading if reported in isolation. Classification algorithms can achieve an accuracy of 95% in a dataset with only 5% of observations belonging to class A by simply classifying all outcomes as class B. It is therefore essential to scrutinize other model metrics such as sensitivity, specificity, PPV and NPV, alongside a confusion matrix of results to allow full appraisal of model performance. Subsampling methods can improve model performance in the case of imbalanced datasets, and although there are potential disadvantages of oversampling, such as increased learning time and the potential for overfitting^34^, in this relatively small dataset learning time was not an issue, and by analysing model performance based on cross-validated prediction results, the risk of overfitting should be ameliorated. There are also arguments around how to judge which model is “best” and ultimately this will depend on the application of the model. This is described in the “No free lunch theorem”^35^ where a point is reached during model tuning where the improvement in one value will be to the detriment of another. In this case, since the algorithm was to provide a clinical decision support tool, the prime performance metrics of interest to veterinary clinicians are likely to be PPV and NPV; i.e. how likely is the prediction of disease to be correct. Final metrics from external validation illustrate clinicians could be 99% confident in a diagnosis of environmental whereas they might only be 86% confident in a diagnosis of contagious origin. A clinician might also be 76% confident a diagnosis of EDP is correct, and 81% confident a diagnosis of EL is correct. The clinician can then interpret the diagnosis in conjunction with the predicted probability of diagnosis as previously discussed, to facilitate an informed decision on whether to act on the predicted diagnosis immediately or pursue further investigations before finalising the diagnosis.

Despite the great potential for ML to assist in medical diagnoses, previous research has reported a risk of a deterioration in diagnostic accuracy on some occasions. For example, in a study of 50 expert clinicians, there was up to a 14% decrease in diagnostic sensitivity when presented with challenging images marked by computer-aided detection^36^. Another study of 30 internal medicine residents showed that the residents exhibited a decrease in diagnostic accuracy (from 57% to 48%) when electrocardiograms were annotated with inaccurate computer-aided diagnoses^37^. Whilst the performance of the models created in this study were robustly validated through cross- and external validation, additional research is required to further validate the effect of ML assisted mastitis diagnosis in practice on diagnostic success and the prevention of mastitis.

There are many available classification algorithms, and in this study the random forest algorithm was found to have the best performance. Previous work assessing 14 different classes of classification algorithm on 115 binary datasets reported that support vector machines, gradient boosting machines and random forests were the three best classifiers^38^, and it has also been shown that random forest classifiers have been able to outperform hundreds of other classification algorithms^20^. One of the advantages of the random forest is the ability to cope with missing data, being able to maintain accuracy when up to 80% of the data are missing^39^. In this study, however, it was found that a large quantity of missing data decreased model performance despite imputation. It was therefore decided to exclude observations with a high degree of missing data and perform random forest imputation on observations with lower numbers of missing data points. Whilst random forest algorithms are able to cope well with missing data, and provide powerful classification accuracy, one disadvantage is their lack of interpretability. Although relative importance of variables within the algorithm can be explored, as shown in the variable importance plot (Fig. 2), it is not possible to directly interpret the magnitude or direction of effect for individual variables using the random forest algorithm^40^.

Cross validation is a robust method of model assessment, and training the algorithm on 90% of the data, and testing on the remaining 10% (repeated 10 times) is relatively robust to prevent overfitting^41^. There remains a possibility, however, of a model overfitting to specific areas within the data, and because of this, an external dataset was randomly selected at the start of the modelling process (i.e. before filtering or pre-processing). The similarity of the EV model performance to that of the CV model indicated that the model was unlikely to be overfitting, and results from this study have a good chance of being applicable for British dairy farms in general.

The ML algorithm identified in this research has the potential to predict an accurate, probabilistic herd mastitis diagnosis which should aid veterinary practitioners in mastitis control. There is great pressure on the agricultural industry to reduce the unnecessary antimicrobial usage (AMU)^42^ and mastitis has been shown to account for up to 68% of all antimicrobial doses in dairy cattle^43^, highlighting the importance of reducing this disease. By reducing the numbers of mastitis cases at herd level by accurate diagnosis of herd mastitis infection patterns and the implementation of effective mastitis control strategies, AMU is likely to be significantly reduced. The application of this methodology in the field of bovine mastitis diagnosis has the potential to aid veterinary clinicians to make a rapid diagnosis, the essential first step in the control of an extremely costly disease in terms of animal health, productivity and welfare.

## Methods

Data used for the study were collated from 1000 dairy farms that had participated in the UK national mastitis control programme, the AHDB Mastitis Control Plan (www.mastitiscontrolplan.co.uk), in an anonymised form. Data available for each farm comprised clinical and sub-clinical mastitis (somatic cell count) records aggregated into 3-month blocks over an 18-month period for lactation numbers 1, >1 and > =1 resulting in 228 features in total (full feature list in *Appendix i*). An expert veterinary herd-level mastitis diagnosis (labelled “diagnosis” in Supplementary Data) had been made for all herds which aligned temporally with the available data. The diagnosis was made by one of three Royal College of Veterinary Surgeons (RCVS) recognised specialists in Cattle Health and Production. In accordance with the national control programme, each herd’s mastitis diagnosis had been classified as either contagious (CONT), environmental dry period (EDP) or environmental lactation period (EL). To make the diagnosis, the veterinary surgeon had access to all farm data, visited the farm on at least two occasions and could perform laboratory tests if deemed suitable. EDP, EL and EDP/EL diagnoses were recoded as ENV for the models to classify a herd mastitis diagnosis as contagious or environmental. EDP vs EL diagnoses were used in a separate model to classify the herd mastitis as being of environmental dry or environmental lactation period origin.

### Data processing

Before analysis, the data were split into a dataset for model tuning and testing using cross-validation analysis (dataset = CV) and an external validation dataset (dataset = EV), each including 50% of farms randomly selected from the original data. The EV dataset was excluded from model construction at the beginning of the research, and models were optimised using repeated tuning and evaluation of the CV dataset. Once model performance was optimised, external validation was conducted using the EV dataset.

Machine learning analysis was performed in R v3.5.1^44^, using the caret package^45^. Farms with incomplete mastitis records (such as missing temporal quarters of data, labelled as “No. Recordings = 0” in Supplementary Material) were excluded from the dataset, as were farms with ≥20% missing features. After omitting farms with large amounts of missing features, any remaining missing data (labelled as NA in Supplementary Material) were imputed using the rfImpute function from the randomForest package^46^. All numeric features were re-scaled to a range of 0–1.

### Model selection, parameter tuning and evaluation of performance

The following machine learning algorithms were evaluated to identify the best predictive model; random forest, gradient boosting machines and support vector machines with radial kernels. For each algorithm, parameter tuning was conducted on the CV dataset using ten-fold cross validation repeated ten times. Random forest^40^ ultimately provided the best model performance as assessed by accuracy, PPV and NPV.

Once the random forest algorithm was identified as the best performer, the model was tuned to optimise performance. *mtry* is defined as the number of variables randomly sampled as candidates at each split^46^ and was initially determined using the number of features (*Var*), *Var/2 and 2Var*, before picking the optimal *mtry* based on the accuracy outcomes^39,47^. Since the contagious diagnosis was a relatively rare event compared with an environmental diagnosis, sensitivity was used to preferentially target this diagnosis in the selection of optimum *mtry* values, whereas accuracy was used to select final *mtry* values for the classification of EDP vs EL (which were relatively even in number). *ntree* was defined as the number of trees to grow^46^ and was set at a default of 500, as it has been shown that the increase in *ntree* does not reliably correlate with increased performance and generally results in no significant gain unless enormous computational power is available^48^.

The probability threshold at which a classification was made was initially set at a standard 0.5. This was explored, and the optimal predictive thresholds determined using the thresholder function^49^, and also assessed visually to optimise model performance. Variable importance was determined for each tree within the random forest by calculating the difference between the prediction accuracy, and the prediction accuracy after a variable had been permuted. This difference was then averaged across all trees and normalised by the standard error to determine the variable importance for each variable^50^. Recursive feature elimination was performed to remove extraneous features and identify the most parsimonious model which maintained optimised performance. Subsampling was performed using up, down and the synthetic minority over-sampling technique (SMOTE)^51^ subsampling methods, however subsampling was not found to improve model performance and was therefore not used to estimate final models.

Cross validation results of both model performance and model tuning options were analysed via a confusion matrix^45^, with models being chosen based on their accuracy, positive and negative predictive values, alongside a variety of performance measures, as has been previously recommended^52,53^. ROC curves and lift plots were also used to analyse models, with calibration plots being used to assess reliability of probability estimates of final models^33^.

## Supplementary information

Appendix i.

Dataset 1.

## References

[1] FAWC. *Opinion on the Welfare of the Dairy Cow*. (2009).

[2] Kossaibati MA, Esslemont RJ (1997). The costs of production diseases in dairy herds in England. Vet. J..

[3] Leslie KE, Petersson-Wolfe CS (2012). Assessment and Management of Pain in Dairy Cows with Clinical Mastitis. Vet. Clin. North Am. Food Anim. Pract..

[4] Hyde RM (2017). Quantitative analysis of antimicrobial use on British dairy farms. Vet. Rec..

[5] Todhunter DA, Smith KL, Hogan JS (1995). Environmental Streptococcal Intramammary Infections of the Bovine Mammary Gland. J. Dairy Sci..

[6] Radostits, O. M., Leslie, K. E. & Fetrow, J. Herd health: food animal production medicine. *Herd Heal. food Anim. Prod. Med*. (1994).

[7] Green MJ, Leach KA, Breen JE, Green LE, Bradley AJ (2007). National intervention study of mastitis control in dairy herds in England and Wales. Vet. Rec..

[8] Down PM, Bradley AJ, Breen JE, Hudson CD, Green MJ (2016). Current management practices and interventions prioritised as part of a nationwide mastitis control plan. Vet. Rec..

[9] Bradley AJ, Green MJ (2004). The importance of the nonlactating period in the epidemiology of intramammary infection and strategies for prevention. Vet. Clin. North Am. Food Anim. Pract..

[10] Bradley, A. *et al*. AHDB Dairy Mastitis Control Plan. 10.1136/vr.j680 (2017). 10.1136/vr.j680 28183899

[11] Guzella TS, Caminhas WM (2009). A review of machine learning approaches to Spam filtering. Expert Syst. Appl..

[12] Koren, Y. *The BellKor Solution to the Netflix Grand Prize*. (2009).

[13] Töscher, A., Jahrer, M. & Bell, R. M. *The BigChaos Solution to the Netflix Grand Prize*. (2009).

[14] Obermeyer Z, Emanuel EJ (2016). Predicting the Future - Big Data, Machine Learning, and Clinical Medicine. N. Engl. J. Med..

[15] Esteva A (2017). Dermatologist-level classification of skin cancer with deep neural networks. Nature.

[16] Fauw, J. D & Ledsam, J. Clinically applicable deep learning for diagnosis and referral in retinal disease. *nature.com* (2018). 10.1038/s41591-018-0107-6 30104768

[17] Gunčar G (2018). An application of machine learning to haematological diagnosis. Sci. Rep..

[18] Barakat, N. & Bradley, P. Intelligible support vector machines for diagnosis of diabetes mellitus. *IEEE Trans. Inf. Technol. Biomed*. **14** (2010). 10.1109/TITB.2009.2039485 20071261

[19] Özçift A (2011). Random forests ensemble classifier trained with data resampling strategy to improve cardiac arrhythmia diagnosis. Comput. Biol. Med..

[20] Fernández-Delgado M, Cernadas E, Barro S, Amorim D, Amorim Fernández-Delgado D (2014). Do we Need Hundreds of Classifiers to Solve Real World Classification Problems?. J. Mach. Learn. Res..

[21] Lynch, C. J. & Liston, C. New machine-learning technologies for computer-aided diagnosis. *Nat. Med*. 1 10.1038/s41591-018-0178-4 (2018). 10.1038/s41591-018-0178-4 30177823

[22] Clifton DA, Niehaus KE, Charlton P, Colopy GW (2015). Health Informatics via Machine Learning for the Clinical Management of Patients. Yearb. Med. Inform..

[23] Fenlon, C. *et al*. A comparison of machine learning techniques for predicting insemination outcome in Irish dairy cows. *Teagasc, Carlow, Irel*. (2016).

[24] Ebrahimie, E., Ebrahimi, F., Ebrahimi, M., Tomlinson, S. & Petrovski, K. R. Hierarchical pattern recognition in milking parameters predicts mastitis prevalence. *Comput. Electron. Agric*. **147**, 6–11 (2018).

[25] Fenlon C (2017). A comparison of 4 predictive models of calving assistance and difficulty in dairy heifers and cows. J. Dairy Sci..

[26] VanderWaal K, Morrison RB, Neuhauser C, Vilalta C, Perez AM (2017). Translating Big Data into Smart Data for Veterinary Epidemiology. Front. Vet. Sci..

[27] Machado G, Mendoza MR, Corbellini LG (2015). What variables are important in predicting bovine viral diarrhea virus? A random forest approach. Vet. Res..

[28] Ducheyne, E. *et al*. *Modelling the spatial distribution of Fasciola hepatica in dairy cattle in Europe. Geospatial Health* **9** (2015). 10.4081/gh.2015.348 25826307

[29] Esener, N. *et al*. Discrimination of contagious and environmental strains of Streptococcus uberis in dairy herds by means of mass spectrometry and machine-learning. 10.1038/s41598-018-35867-6 10.1038/s41598-018-35867-6 PMC6269454 30504894

[30] Ebrahimi, M., Mohammadi-Dehcheshmeh, M., Ebrahimie, E. & Petrovski, K. R. Comprehensive analysis of machine learning models for prediction of sub-clinical mastitis: Deep Learning and Gradient-Boosted Trees outperform other models. *Comput. Biol. Med*. **114** (2019). 10.1016/j.compbiomed.2019.103456 31605926

[31] Ebrahimie E, Ebrahimi F, Ebrahimi M, Tomlinson S, Petrovski KR (2018). A large-scale study of indicators of sub-clinical mastitis in dairy cattle by attribute weighting analysis of milk composition features: highlighting the predictive power of lactose and electrical conductivity. J. Dairy Res..

[32] Sharifi, S. *et al*. Integration of machine learning and metaanalysis identifies the transcriptomic bio-signature of mastitis disease in cattle. *Plos One* **13** (2018). 10.1371/journal.pone.0191227 PMC5823400 29470489

[33] Vuk, M. & Curk, T. *ROC Curve, Lift Chart and Calibration Plot. Metodološki zvezki* **3** (2006).

[34] Weiss, G. M., Mccarthy, K. & Zabar, B. *Cost-Sensitive Learning vs. Sampling: Which is Best for Handling Unbalanced Classes with Unequal Error Costs?*

[35] Wolpert, D. H. & Macready, W. G. *No Free Lunch Theorems for Optimization*. (1996).

[36] Povyakalo AA, Alberdi E, Strigini L, Ayton P (2013). How to Discriminate between Computer-Aided and Computer-Hindered Decisions. Med. Decis. Mak..

[37] Tsai TL, Fridsma DB, Gatti G (2003). Computer Decision Support as a Source of Interpretation Error: The Case of Electrocardiograms. J. Am. Med. Informatics Assoc..

[38] Wainer, J. *Comparison of 14 different families of classification algorithms on 115 binary datasets*. (2016).

[39] Breiman, L. & Cutler, A. Manual–Setting Up, Using, And Understanding Random Forests V4.0. (2003).

[40] Breiman L (2001). Random Forests. Mach. Learn..

[41] Ron Kohavi. A study of cross-validation and bootstrap for accuracy estimation and model selection. *Proc. 14th Int. Jt. Conf. Artif. Intell. - Vol. 2 2077* (1995).

[42] O’Neill, J. Antimicrobials in agriculture and the environment: reducing unnecessary use and waste the review on antimicrobial resistance. (2015).

[43] Kuipers A, Koops WJ, Wemmenhove H (2016). Antibiotic use in dairy herds in the Netherlands from 2005 to 2012. J. Dairy Sci..

[44] Team, R. C. R: A Language and Environment for Statistical Computing. (2018).

[45] Kuhn., M. *et al*. caret: Classification and Regression Training. R Packag. (2018).

[46] Liaw A (2018). randomForest: Breiman and Cutler’s Random Forests for Classification and Regression. R Packag..

[47] Liaw, A. & Wiener, M. Classification and Regression by randomForest. *R News* (2002).

[48] Oshiro, T. M., Perez, P. S. & Baranauskas, J. A. How Many Trees in a Random Forest? In 154–168 (Springer, Berlin, Heidelberg, 10.1007/978-3-642-31537-4_13 2012).

[49] Kuhn, M. Classification and Regression Training. (2018).

[50] Kuhn, M. *Variable Importance Using The caret Package*. (2011).

[51] Chawla NV, Bowyer KW, Hall LO, Kegelmeyer WP (2002). SMOTE: Synthetic Minority Over-sampling Technique. J. Artif. Intell. Res..

[52] Branco P, Torgo L, Ribeiro RP (2016). A Survey of Predictive Modeling on Imbalanced Domains. ACM Comput. Surv..

[53] Sokolova M, Lapalme G (2009). A systematic analysis of performance measures for classification tasks. Inf. Process. Manag..

