# Supplementary material for: Automated prediction of mastitis infection patterns in dairy herds using machine learning
Source: Sci Rep. 2020 Mar 9;10:4289. doi: 10.1038/s41598-020-61126-8 (PMC7062853; doi:10.1038/s41598-020-61126-8)
Supplement: Supplementary file 1 — Appendix i. [file 41598_2020_61126_MOESM1_ESM.pdf]

Automated prediction of mastitis infection patterns in dairy herds using machine learning

Robert M. Hyde[*1], Peter M. Down[*], Andrew J. Bradley*†, James E. Breen*, Chris Hudson[*], Katharine A. Leach†, Martin J. Green[*]

[*]School of Veterinary Medicine and Science, University of Nottingham, Sutton Bonington Campus, Leicestershire, United Kingdom LE12 5RD

†Quality Milk Management Services, Cedar Barn, Easton Hill, Wells, BA5 1DU

[1]Corresponding author: Robert M. Hyde

Email: Robert.hyde1@nottingham.ac.uk

Feature	*Label in supplementary data table*	*Mean (range) of Lactation >=1*
Date of 3-month period	Quarter date	
Number of recordings per 3-month period	No. Recordings	3 (0-7)
Bulk milk somatic cell count (‘000 cells/ml)	BMSCC ('000 cells/ml)	219 (0-1486)
% of herd with individual somatic cell count >200,000 cells/ml for >= 2 consecutive months	% Chronic	15 (0-75)
% of herd with individual somatic cell count >200,000	% >200K	23 (0-80)
% of herd with new somatic cell count >200,00 cells/ml >30 days in milk	Lactation New IMI(%)	10 (0-56)
% of herd with new somatic cell count >200,00 cells/ml <=30 days in milk	Dry Period New IMI(%)	18 (0-100)
% of herd with somatic cell count >200,000 before drying off that had subsequent somatic cell count <= 200,000 at first milk recording post-calving	Apparent' Dry Period Cure(%)	70 (0-100)
Incidence rate of clinical mastitis cases <=30 days in milk (per 12 cows)	Dry Period Origin CM Rate (in 12)	1 (0-13)
Incidence rate of clinical mastitis cases >30 days in milk (per 12 cows)	Lactating Period Origin CM Rate (in 12)	2 (0-17)
Incidence rate of cow quarter level clinical mastitis cases (per 100 cows/year)	Qrt CMIR (/100 cows/year)	53 (0-354)
Incidence rate of cow level clinical mastitis cases (per 100 cows/year)	Cow CMIR (/100 cows/year)	47 (0-309)
Clinical mastitis cure rate (for first cases)	Clinical Mastitis Cure Rate (1st Case)	42 (0-200)
Number of cows calved	No. Calved	52 (0-586)

Appendix i: Original features available for each of the 6, 3-month periods. Each of these parameters (other than date and number of recordings) was available for both heifers (lactation 1 animals, labelled as L=1), cows (lactation >1 animals, labelled as L>1), and cows and heifers combined (lactation >= 1 animals,

labelled as L>=1) for each of the 6, 3-month periods (labelled as Q0 for the most recent 3-month period, Q-1 for the previous 3-month period, Q-2 for the 3-month period before that etc). “Diagnosis” describes the specialist diagnosis of herd level mastitis origin.
